# The nitrogen isotopic composition of biotic components in Northern Biscayne Bay and Associated Waterways, South Florida USA: implications for sources of nitrogen

**DOI:** 10.1007/s11356-026-37702-2

**Published:** 2026-04-07

**Authors:** Peter K. Swart, Amel Saied, Sean P. Ahearn, Tiffany Troxler, Maribeth Gidley, Christopher Sinigalliano, Aliza Karim, Elizabeth Kelly, Rachel Silverstein

**Affiliations:** 1 University of Miami, Rosenstiel School of Marine, Atmospheric, and Earth Sciences, 4600 Rickenbacker Causeway, Miami, FL 33149 USA; 2 Research & Development, Beta Analytic Inc., 4985 S.W. 74Th Court, Miami, FL 33155 USA; 3 Earth and Environment Department and Institute of Environment, Florida International University, Miami, FL 33199 USA; 4 Cooperative Institute for Marine and Atmospheric Studies, University of Miami, 4600 Rickenbacker Causeway, Miami, FL 33149 USA; 5 NOAA Atlantic Oceanographic and Meteorological Laboratory, Ocean Chemistry and Ecosystems Division, 4301 Rickenbacker Causeway, Miami, FL 33149 USA; 6 Miami Waterkeeper, P.O. Box 141596, Coral Gables, FL USA

**Keywords:** Nitrogen isotopes, Nutrients, Sewage, Algae, Storm water, Canals, Biscayne Bay

## Abstract

Over an approximate twelve-month period, between June 2021 and June 2022, the δ^15^N values of aquatic vegetation and sediments and the δ^18^O and δ^15^N values of the NO_3_^−^ in water samples were analyzed in order to understand the sources and processes affecting nitrogen entering the northern portion of Biscayne Bay, an urban area located to the west of Miami, in the southern United States. Samples were collected from 22 sites, primarily located in waterways feeding into the Bay (Miami River, Little River, Biscayne Canal, and Snake Creek). Additional samples were also collected in the central region of Biscayne Bay, around Virginia Key, Key Biscayne, Coconut Grove, and Matheson Hammock. The highest δ^15^N values were found in algae and plants collected from the Biscayne Canal, Little River, and Miami River, inland sites heavily influenced by land-based source of pollution such as storm water, septic tanks, and industrial runoff, while lower values were measured at the central Biscayne Bay sites. The Little River sites showed a strong seasonality with δ^15^N values of the vegetation ranging from + 8‰ in the wet season to over + 13‰ at the start of the dry season, while the Miami River and Biscayne Canal sites exhibited δ^15^N values of between + 9 to + 13‰ throughout the year. The elevated δ^15^N values are associated with the input of anthropogenic waste, presumed to originate from septic tanks along the canals. Elevation of δ^15^N values in excess of typical values found in humans is driven by fractionation of nitrogen during assimilation, nitrification, denitrification, volatilization of NH_4_^+^ and anaerobic ammonium oxidation in septic tanks with clogged drain fields. We propose that dissolved inorganic nitrogen, derived from septic tanks with elevated δ^15^N values, mixes with nitrogen derived from the natural environment as well as that sourced from artificial fertilizers (both with lower δ^15^N values) to produce pools of nitrogen with intermediate δ^15^N values. These are then further enriched in ^15^N during the process of assimilation and nitrification which fractionates the δ^15^N value of the residual NO_3_^−^. At all locations there is close to a 1:1 relationship between the δ^15^N and δ^18^Ο values of the nitrate, expected as a result of fractionation during assimilation. Mixing between natural sources of nitrogen, or nitrogen arising from anthropogenic sources or other animal waste and nitrogen arising from contamination by fertilizer produces a positive association between NO_3_^−^ concentration and δ^15^N_NO3-_ values at some locations.

**Supplementary Information:**

The online version contains supplementary material available at 10.1007/s11356-026-37702-2.

## Introduction

The ^15^N/^14^N ratio of inorganic and organic nitrogen has been widely used to explore anthropogenic inputs in both marine and freshwater aquatic ecosystems (Alldred et al. 2024; Costanzo et al. 2005; Sammarco et al. 1999; Zhang et al. 2022) and others. The premise of such studies is that humans, by virtue of their higher trophic position, produce nitrogenous waste elevated in ^15^N which is in turn recorded in the δ^15^N values of the water (inorganic nitrogen, particulate and dissolved organic material) and biota within an affected ecosystem. While δ^15^N values as low as + 5 ‰ have been suggested to be indicative of anthropogenic nitrogen (Lapointe et al. 2005), there is debate as to not only what the threshold δ^15^N values for anthropogenic influences are, but also to the extent that variations in the ^15^N/^14^N ratio are also caused by fractionation during processes such as assimilation, nitrification, and denitrification that affect the organic and inorganic nitrogen species in the ecosystem (Guo et al. 2022; Swart et al. 2013). This study attempts to identify the importance of both the source and the processes that influence the concentrations of inorganic nitrogen in water currently entering the northern portion of Biscayne Bay, located in southern Florida USA, using the δ^15^N and δ^18^O values of NO_3_^−^ as well as the δ^15^N values of sedimentary organic material and plants and algae. Northern Biscayne Bay is an ideal location for such a study as it is located adjacent to a large urban population, a significant proportion of which use septic systems to process waste. Such a location leaves little doubt that the δ^15^N values will be influenced by sewage and hence variations from mixing patterns will provide information on the fractionation processes mentioned earlier. In addition to being an important site to study competing influences of source and process on the δ^15^N values, Biscayne Bay is a vital component of a sub-tropical ecosystem responsible for the development of numerous juvenile vertebrate and invertebrate species that populate the northern portion of the Florida Reef Tract, the largest barrier reef in the contiguous United States and therefore understanding the sources of nitrogen loading in the Bay is of considerable importance. To accomplish these goals, the stable nitrogen isotope dynamics of algae and aquatic plants and the oxygen and nitrogen isotopic systematics of the nitrate in water were investigated in 22 sites within the water shed of N. Biscayne Bay over ~ 12-months. While other chemical and biological data were also collected at the same time, including standard nutrient parameters (temperature, salinity, total nitrogen, soluble reactive phosphorus, NO_3_, NH_4_^+^, chlorophyll, turbidity, and dissolved oxygen content), and various biological indices of pollution (fecal indicator bacteria and genetic markers of the fecal activity of humans and dogs), this paper deals principally with the nitrogen isotopes of the vegetation and waters. The additional data are summarized in a report submitted to the Environmental Protection Agency (EPA) (Silverstein et al. 2024) and have been submitted to the appropriate databases. These data are referenced where appropriate.

### Background

**Fig. 1:**
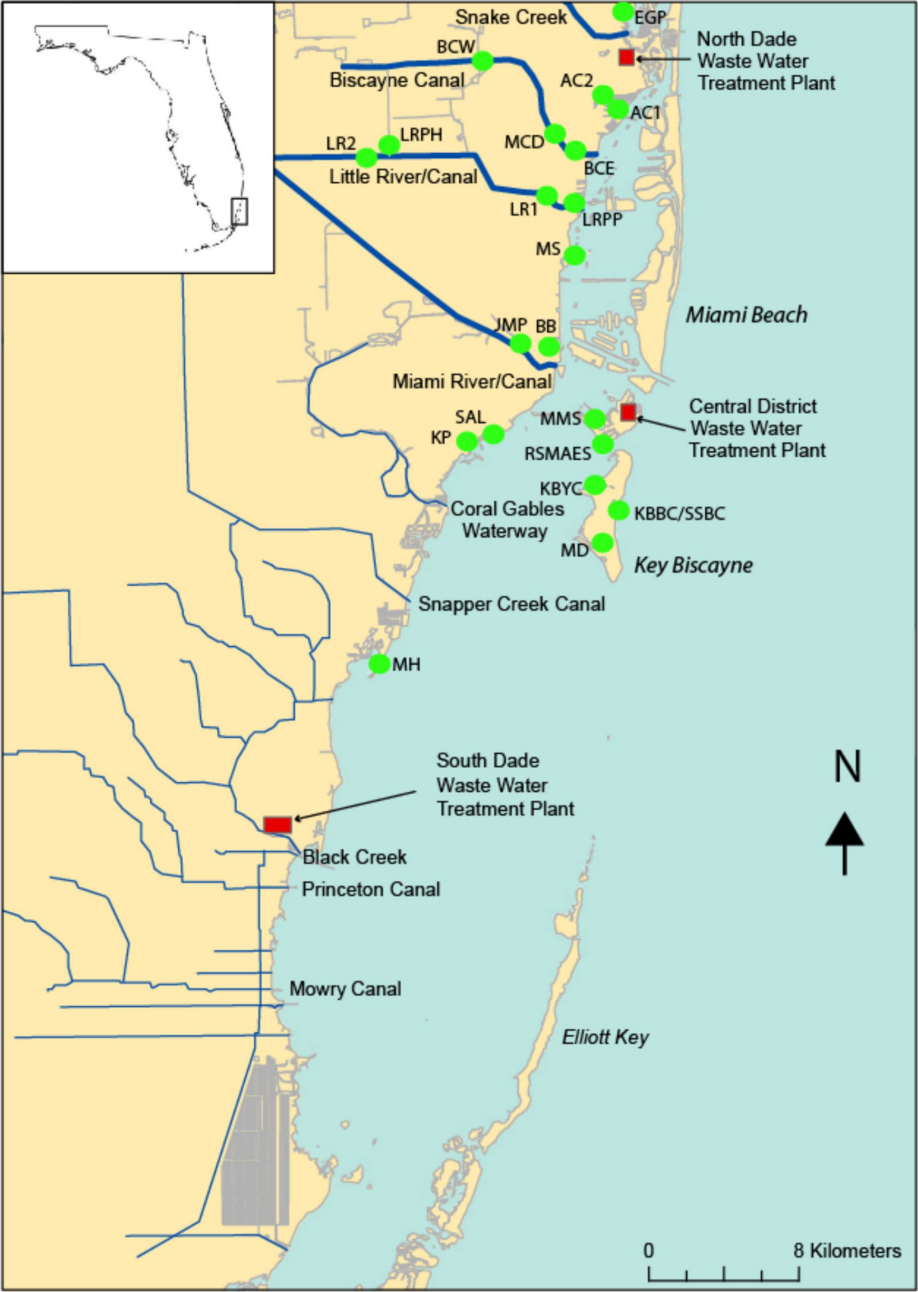
Location map of Biscayne Bay and the 22 stations (green dots) used in this study; insert shows the region relative to the state of Florida. The IDs of each of the sites are given in Table 1. Also shown are the various canals sampled (Little River, Biscayne Canal, Arch Creek, and the Miami River)

**Fig. 2:**
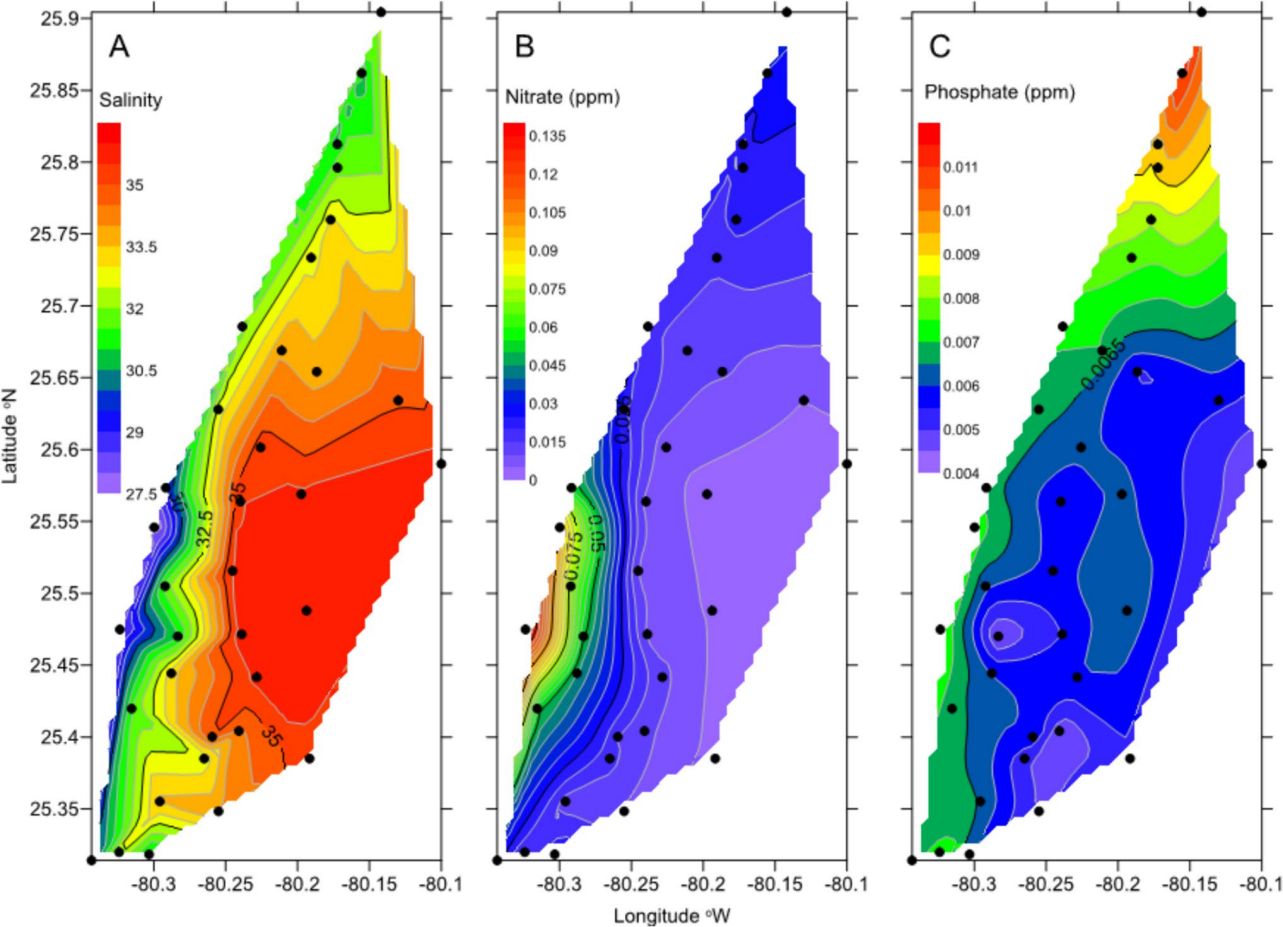
Average (**A**) salinity, (**B**) NO_x_, and (**C**) PO_4_^3−^ in waters collected from Biscayne Bay between 1993 and 2008 from 36 stations (data from FIU database). Salinities are the lowest and NO_x_ concentrations the highest in the southern portion of the Bay associated with discharge from Mowry, Princeton, and Military canals. Concentrations of phosphate are generally higher in the northern portion of the Bay

Biscayne Bay is a partially enclosed body of water located immediately to the east of the city of Miami, an urban area of ~ 2.7 million people (Fig. 1). In the northern portion of the Bay, mangroves, that would have originally lined the coast, have been largely removed, and urban development extends to the coastline, with the central portions of the Bay being occupied by artificial islands composed of locally derived dredge material. Variations in the salinity arise from the mixing of rainwater, seawater, and freshwater emanating from canals and groundwater. Saltwater intrusion and water levels in adjacent canals and water conservation areas and ultimately Lake Okeechobee are controlled by the South Florida Water Management District (SFWMD) by means of water control structures, usually located near or at the coastline. While the range of salinity in Biscayne Bay is well represented by monthly measurements at 36 locations in the bay made between 1993–2008 (Caccia and Boyer 2005) (Fig. 2A), the present salinity balance in southern Biscayne Bay is probably significantly different to that 150 years ago, when there are reports that there were abundant fresh-water springs resulting from the high hydrological head of freshwater on the mainland (Kohout 1966; Munroe and Gilpin., 1990). In the south, present freshwater input is derived from direct precipitation (53%), with smaller quantities emanating from canals (37%), and groundwater (10%) (Stalker et al. 2009). Less is known about the history of salinity in the northern portion of the bay, although it is probably significantly more saline than before an inlet was created in 1925 that allowed saltwater to directly enter the very northern portion (Haulover Inlet) from the Straits of Florida. Groundwater inputs into the northern portion of the bay are at the moment unknown, although it is possible that extensive dredging activity that has taken place in this area during the construction of artificial islands during the early portion of the twentieth century could have penetrated into the Biscayne Aquifer and allowed fresh groundwater to affect the salinity of the Bay.

Nutrient analyses of the water samples, previously analyzed for salinity, show high concentrations of dissolved inorganic nitrogen (DIN) close to the coast (up to 20 μM), in the southern portion of the bay (Caccia and Boyer 2007), but lower concentrations (1–4 μM) in the north (Fig. 2B). Such high concentrations contrast with ranges of between 0.1 to 0.5 μM on the Florida reef tract (Szmant and Forrester 1996). While the high concentrations of DIN in the south have been interpreted as reflecting nutrients mainly derived from agricultural runoff and input via canals, rather than from other anthropogenic sources (Caccia and Boyer 2007), studies using stable N isotopes of algae and sea grasses indicate that these high concentrations are associated with elevated δ^15^N values in algae and seagrasses, thus perhaps suggesting an anthropogenic origin. Concentrations of phosphate show the opposite pattern to nitrate, with southern Biscayne Bay showing lower total phosphate concentrations than in the north (Fig. 2C).

### Stable nitrogen and oxygen isotopic values

The ratio of ^15^N to ^14^N are reported relative to a standard in parts per thousand as a δ^15^N value. In this scheme, common to all stable isotopes, a value of zero indicates that a substance has the same ^15^N/^14^N ratio as the standard, in the case, atmospheric nitrogen. A positive value indicates more ^15^N in the sample and a negative value less ^15^N. Recycled nitrogen enters the biological cycle through the decay or organic material, whereas new nitrogen arises from either through nitrogen fixation or through the addition of man-made fertilizers produced by the Haber–Bosch process. Nitrogen compounds form by these processes result in minimal fractionation so that the δ^15^N values of nitrogen fixers and commercially produced crops have δ^15^N values close to zero (Bateman and Kelly 2007; Denk et al. 2017; Hoering and Ford 1960). Herbivores become enriched in the heavier isotope of nitrogen as a result of the excretion of nitrogenous waste that contains less ^15^N with the amount of enrichment being ~ 3- 4 ‰ per trophic level (DeNiro and Epstein 1981; Schoeninger and DeNiro 1984). This process continues in each trophic level so that humans typically have δ^15^N values of between ~  + 8 to + 12 ‰, the precise value being dependent upon diet (Bird et al. 2021; Bowen et al. 2009). Once vegetative and animal waste decays, it is converted to ammonia, with minimal change in isotopic composition, and then to nitrite and nitrate (nitrification). Although a significant amount of fractionation accompanies nitrification (Cifuentes et al. 1989), in systems where there is a large amount of NH_4_^+^ available, oxidation of NH_4_^+^ becomes the limiting step leading to high δ^15^N values in the residual NH_4_^+^ (Kendall et al. 2008). Both NO_3_^−^ and NH_4_^+^ are utilized by plants, a process known as assimilation that results in a small amount of fractionation so that the plant preferentially utilizes ^14^N and leaves ^15^N behind (Granger et al. 2010). At low concentrations fractionation is minimal, increasing in the presence of higher amounts of NO_3_^−^ (Swart et al. 2014). In anoxic environments, the NO_3_^−^ can be utilized as an electron acceptor (denitrification), a process that results in significant amount of fractionation with the residual pool becoming enriched in ^15^N and the produced N_2_ or NO_2_ gas containing less ^15^N (Cline and Kaplan 1975). All these process as well as the anaerobic oxidation of NH_4_^+^ and NO_2_^−^ (Anammox) produce variability in the δ^15^N values of organic material and inorganic nitrogen unrelated to that of the original source (Brunner et al. 2013). A typical example might be the continual assimilation of NO_3_^−^ present in a body of water, as exemplified in laboratory experiments (Swart et al. 2014). In these experiments macroalgae were grown in an incubator using a stock solution of NaNO_3_ with an initial δ^15^N value of + 3 ‰. Over a period of 48 h, the concentration of NO_3_^−^ in the 60 μM experiment decreased to 1 μM while its δ^15^N value increased to + 9.5 ‰. In addition to the δ^15^N values of nitrate, its δ^18^O value can also be used to trace the source and fate of NO_3_^−^ in aquatic environments (Aravena and Robertson 1998; Casciotti 2009; Kendall 1998; Knapp et al. 2005, 2008a, b; Sigman et al. 2009; Wankel et al. 2006, 2009). Nitrate that is derived or transformed by different sources or processes, produces varying relationships between the δ^15^N and the δ^18^O values. During assimilation of nitrate by algae in the example just described, ^15^N and ^18^O are fractionated to the same extent leading to an approximate 1:1 correlation (Swart et al. 2014), thus allowing the processes, responsible for elevating the δ^15^N values, to be identified. However, while processes such assimilation, denitrification, and nitrification change the relationship between the δ^15^N and δ^18^O values (Kendall et al. 2008), mixing between sewage, fertilizer, and atmospheric derived nitrate complicates the interpretation of the data.

## Methods

**Fig. 3:**
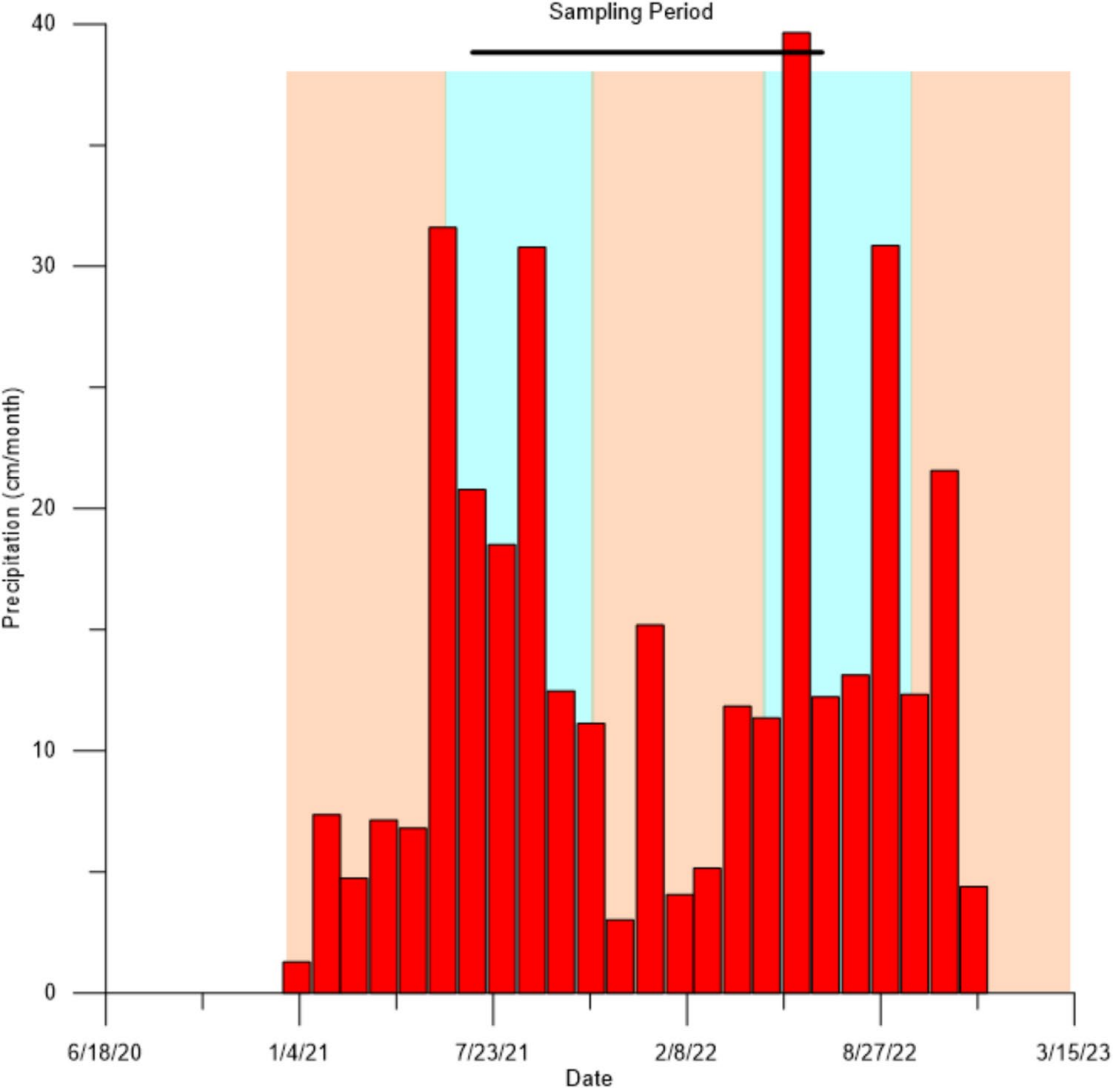
Precipitation measured at Miami International Airport in Miami between 2021 and 2022. Shaded areas represent the wet (blue) and dry (orange) seasons with the wet season officially lasting between ~ May 15 and ~ October 15 of each year. The sampling period lasted from June 2021 to June 2022

Small amounts of the recent growth of algae and benthic plants were collected approximately every two weeks, between June 2021 and June 2022, from 22 locations mainly within Northern Biscayne Bay and adjacent waterways/canals (Table 1 and Fig. 1). The actual sampling dates are included in the supplemental material (Table S1). The collection of material was intended to sample the canals on (1) the falling tide in order to maximize the possibility of capturing samples of land-based pollutants, and (2) to sample both the wet and dry seasons in South Florida. The wet season in South Florida stretches from May 15 to October 15 of each year, during which between 65 to 75% of the annual precipitation occurs. The annual rainfall measured at Miami International Airport during 2021 and 2022, was 155.6 and 181.7 cm/yr, respectively, slightly more than the annual average of 150 cm/yr, with the patterns of rainfall during these years conforming to the expected wet and dry seasons, with 69% of the rainfall falling in the 2021 wet season and 59% in the 2022 wet season (Fig. 3). As a result of the geographical dispersion of the sites, sampling was usually carried out over two days. Because samples were collected from both marine and freshwater habitats, it was not possible to collect the same species from all sites and therefore at each site a range of different species were sampled so an assessment could be made as to whether different species were recording similar variations in the δ^15^N values. The following species were collected, where present, in the marine and brackish habitats; *Avrainvillea* sp*, Caulerpa* sp.*, Cladophora* sp*., Codium* sp, *Dasycladus* sp*., Dichtoyta* sp.*, Jania* sp*, Laurencia* sp*, Penicillus* sp*., Thalassia testudinum, Halodule* sp*., Syringodium* sp*., Ulva* sp*., Halimeda* sp*., Sargassum* sp*.,* and filamentous green algae*.* In the freshwater environments *Hydrilla* sp*., Vallisneria* sp*.* and filamentous green algae growing on shells, mangroves, and rocks were sampled. In total ~ 1,100 samples of plants were collected.

**Table 1:** Names of Sites, latitude and longitude, and watersheds; CG = Coconut Grove, KB = Key Biscayne, CBB = Central Biscayne Bay, MR = Miami River, LR = Little River, MNBB = Middle north Biscayne Bay, BC = Biscayne Canal, AC = Arch Creek, NBB = North Biscayne Bay. Also shown are the mean salinity and inorganic nitrogen measured during the study

Site	Code	Watershed	Latitude	Longitude	Salinity	NO3-	NH4 +
			^o^N	^o^W	g/kg	µM	µM
Arch Creek 1	AC1	NBB	25.9059	80.1638	5.6	7.1	63.3
Arch Creek 2	AC2	NBB	25.8969	80.1588	22.0	6.7	21.0
Biscayne Canal East	BCE	BC	25.8717	80.1688	16.3	7.7	8.5
Biscayne Canal West	BCW	BC	25.9163	80.2238	0.2	7.4	4.4
Brickell Bay Drive	BB	MR	25.7580	80.1891	31.3	2.2	1.5
East Greynolds Park	EGP	NBB	25.9294	80.1512	14.8	4.3	6.0
Jose Marti Park	JMP	MR	25.7715	80.2003	12.5	8.7	6.6
Kennedy Park	KP	CG	25.7340	80.2268	30.7	1.3	1.4
Key Biscayne Beach Club	KBBC	KB	25.6950	80.1570	32.8	0.6	2.4
Key Biscayne Yacht Club	KBYC	KB	25.6999	80.1690	33.0	1.1	1.2
Little River 1	LR1	LR	25.8502	80.1886	4.0	18.5	12.2
Little River 2	LR2	LR	25.8710	80.2420	0.3	21.4	12.1
Little River Pelican Harbor	LRPH	LR	25.8716	80.2348	0.2	7.6	21.8
Little River Pocket Park	LRPP	LR	25.8460	80.1770	12.6	15.8	10.9
Mariner Drive	MD	KB	25.6829	80.1656	32.4	1.7	4.2
Matheson Hammock	MH2	CG	25.6700	80.2560	29.2	1.0	3.9
Miami Country Day	MCD	BC	25.8739	80.1821	2.4	7.7	7.2
Miami Marine Stadium	MMS	CBB	25.7420	80.1680	32.7	0.8	0.9
Morningside Park	MS	MNBB	25.8233	80.1783	29.1	2.6	1.7
Rosenstiel School, UM	RSMAES	CBB	25.7310	80.1620	34.0	1.5	0.9
Shake-A-Leg	SAL	CG	25.7313	80.2331	30.4	1.6	3.2
Silver Sands Beach Resort	SSBR	KB	25.6950	80.1570	33.3	0.5	3.9

### Benthic samples

As samples were returned to the Rosenstiel School of Marine, Atmospheric, and Earth Sciences (RSMAES) at the University of Miami (Fig. 1), they were sorted, identified, and separated by genus and/or species, rinsed in distilled water, and dried at 40 °C for approximately 4–7 days. Dried material was ground using a Wiley Mill to a size of < 40 μm. Approximately 1–4 mg of this material was weighed and used for the analyses of δ^15^N values. The samples were not acidified to remove any carbonate as an overwhelming amount of previous work, within our own laboratory and elsewhere, has shown this procedure significantly alters the δ^15^N values or organic material derived from both plants and animals, particularly in fresh material (Brodie et al. 2011a, 2011b; Lohse et al. 2000). The lack of acidification meant that some samples were contaminated by carbonate material and while the δ^13^C values and CN ratios have not included in this paper they are submitted to the Earthchem database (See Declarations sections of this paper).

### Sedimentary organic material

Sediment samples were collected on several occasions during the sampling period. As a result of access limitations, sediment samples were not collected from site AC2. All collected samples were freeze-dried upon return to RSMAES and then ground with a mortar and pestle.

### Dissolved inorganic nitrogen (nitrate) concentration and isotopic measurements

Sample aliquots (10 to 40 mL) were filtered through 0.2 μm Isopore polycarbonate filters (Millipore Sigma, Burlington, MA) into acidified (0.2 mL of 18% HCl) 40 mL amber vials. The concentration of NO_3_^−^ was measured (EPA Method 353.2) (EPA 1993) using an AQ300 Discrete Analyzer (Seal Analytical, Inc., Mequon, WI). A limited number of water samples (*n* = 70) containing more than ~ 2.0 µM (N-NO_3_^−^) were then analyzed for their δ^15^N_NO3-_ and δ^18^O_NO3-_ values using a chemical reduction method. In this method NO_3_^−^ in solution is reduced to nitrous oxide (N_2_O) which is then analyzed using continuous flow (CF) on a Delta V Isotope Ratio Mass Spectrometer (IRMS, Thermo Fisher Scientific, Waltham, MA) using a Gas Bench II system fitted with a denitrification kit (Altabet et al. 2019; Foreman et al. 2016) at Beta Analytical in Miami Fl. Each sample analysis was referenced to 10 injections of N_2_O, yieling a standard deviation of < 0.1‰ for δ^15^N-NO_3_^−^ and δ^18^O-NO_3_^−^. The reference N_2_O gas was standardized using international nitrate isotopic reference materials (United States Geological Survey) USGS-32, USGS-34, and USGS-35 (Coplen 2021). Method uncertainty was ± 2‰ (1 RSD) for δ^18^O-NO_3_^−^ and ± 0.5‰ (1 RSD) for δ^15^N-NO_3_^−^, comparable to previous analyses (Sigman et al. 2009).

### Analytical

Elemental and isotopic abundances for solid materials were determined using a Costech elemental analyzer interfaced to a stable isotope mass spectrometer (Thermo Fisher Delta V) at the University of Miami. Analytical reproducibility for solid materials is 0.2‰ for δ^15^N. Average values for each sample were determined and the standard deviation calculated. Samples that had standard deviations which were greater than two standard deviations from the mean were reanalyzed and the average values of the two or more samples used.

### Standards

The isotopic values of δ^15^N_NO3-_ and δ^18^O_NO3-_ are reported relative to N_2_ in air, and Vienna Standard Mean Ocean Water (V-SMOW).

### Statistics

Correlations between variables were calculated using a Pearson correlation coefficient unless datasets contained less than 20 individuals, in which case a Spearman’s rank correlation coefficient was used. Contour maps were constructed using a Kriging routine in Surfer 9.0 that generates an interpolated grid based on estimates derived from a sampled dataset (Cressie 1990; Isaaks et al. 1989).

## Results

### Algae and plants

**Fig. 4:**
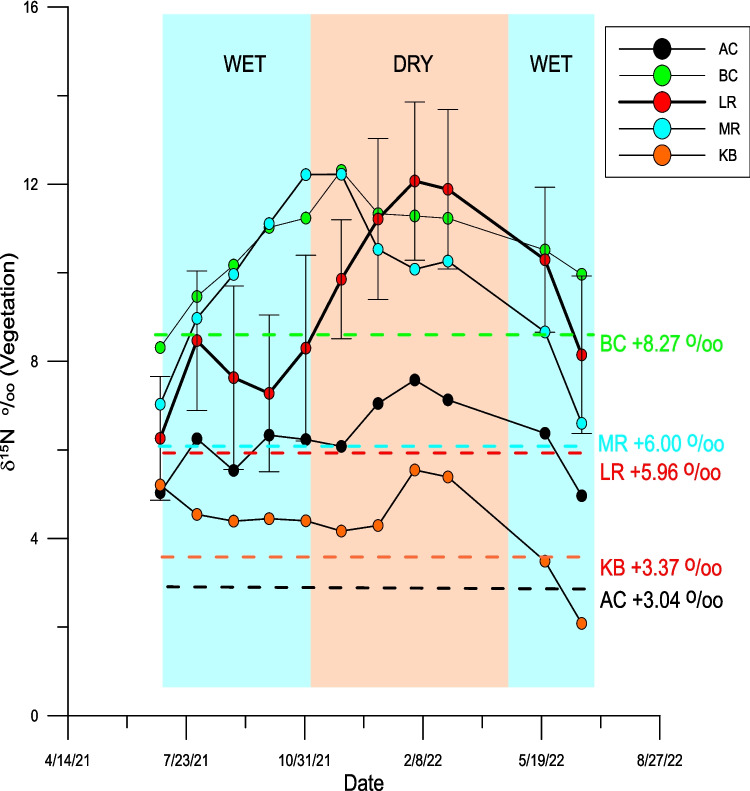
Time series of monthly δ^15^N_VEG_ values for selected watersheds (LR = Little River, AC = Arch Creek, BC = Biscayne Canal, JMP = Jose Marti Park, KB = Key Biscayne). Error bars represented the standard ± 1 standard deviation for the Little River sites. Standard deviations are not shown for all sites for reasons of clarity. The average δ^15^N values of the organic material in the sediments for each watershed are shown by the dashed line. Similar plots are shown for all ‘watersheds’ in the supplemental material

The mean and standard deviation of the salinity and nutrient concentrations measured from the various locations are summarized in Table 1. While all the raw isotopic data are included in the supplemental material (Table S1), the monthly mean δ^15^N values for the vegetative components are shown in Table 2. Other samples excluded from the average values calculated at each site for each collection period were *Sargassum fluitans and S. natans* (planktonic species that are blown into Biscayne Bay) and mangrove detritus (a terrestrial species). Considering all the remaining vegetation samples, the average δ^15^N value is + 7.1 ‰ and range between −2.66 and + 23.36‰. As it is possible that some of the variations in the δ^15^N values could reflect differences between the various species analyzed, multiple species of algae and plants were analyzed from sites where multiple species were present and the δ^15^N values of the different species correlated with one another. For example, the δ^15^N values of the freshwater species collected, i.e., Eelgrass (*Vallisneria* sp.) and Water Thyme (*Hydrilla* sp.), correlate strongly with the δ^15^N values of filamentous green algae collected in the same habitat (Fig. S4-1). In marine environments, the δ^15^N values of the green algae correlated well with that of Manatee grass (*Syringodium* sp*.*) and brown (excluding *Sargassum* sp) and red algae (Fig. S4-2). In contrast, the δ^15^N values of Shoal grass (*Halodule* sp) and Turtle grass (*Thallassia* sp*.*) were not strongly correlated with either Manatee grass or green, red, and brown algae (Fig. S4-3). For this reason, Shoal and Turtle grasses are not included in the calculation of the mean values of the vegetation. During each month there were multiple trips to the sample sites and therefore, in order to make the data more manageable, all data for a given month were averaged to give one data point for the δ^15^N_veg_ values for each month for each site. For operational reasons, samples were not retrieved from three sites in August 2021, two in December 2021, and one in March 2022. For these locations the mean value calculated from adjacent months was substituted for graphing purposes only. Considering the number of sites and the number of months this yielded 242 potential data points. The spatial patterns of δ^15^N_veg_ values are shown in Figure S4-4 and separated into the wet (May–October) and dry (November–April) seasons in Figure S4-4B and S4-4C. The monthly values for five groups of sites are shown in Fig. 4 as a function of wet and dry season. In the case of all sites the most positive δ^15^N values are shown either in the dry season or at the end of the wet season. The δ^15^N for all samples analyzed, including species not included in the calculations of the mean values, are contained in the supplemental material. Contour plots showing temporal variations for each site are included in the supplemental material (Figures S4-5 to S4-19).

**Table 2:**
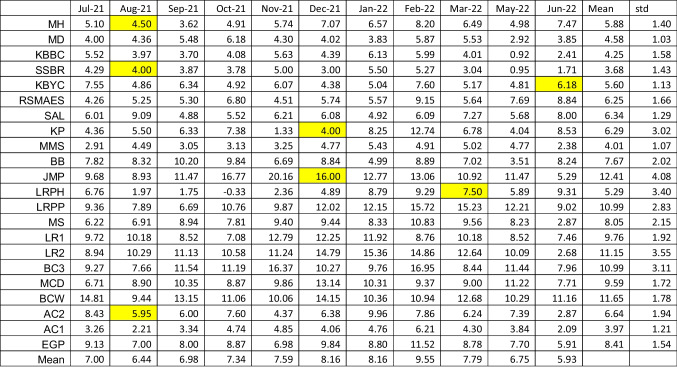
The δ^15^N values (‰) and standard deviations of yearly values of the mean vegetative components. The mean standard deviation is 2.1 and varies between 0.3 and 4.7‰. The yellow shaded boxes are sites that were not sampled during that particular month and values are the averages of adjacent months collected from the same site

### Planktonic algae

All of the algae sampled in this study were benthic with the exception of *Sargassum natans* and *S. fluitans,* which are planktoni*c*. One specimen of the benthic variety of Sargassum, *S. pteropleuron* was sampled at Matheson Hammock*.* The planktonic variety of Sargassum, possessed mean δ^15^N value of + 2.34 (*n* = 45), while the benthic variety had a mean δ^15^N value of + 10‰.

### Sedimentary organic material

**Fig. 5:**
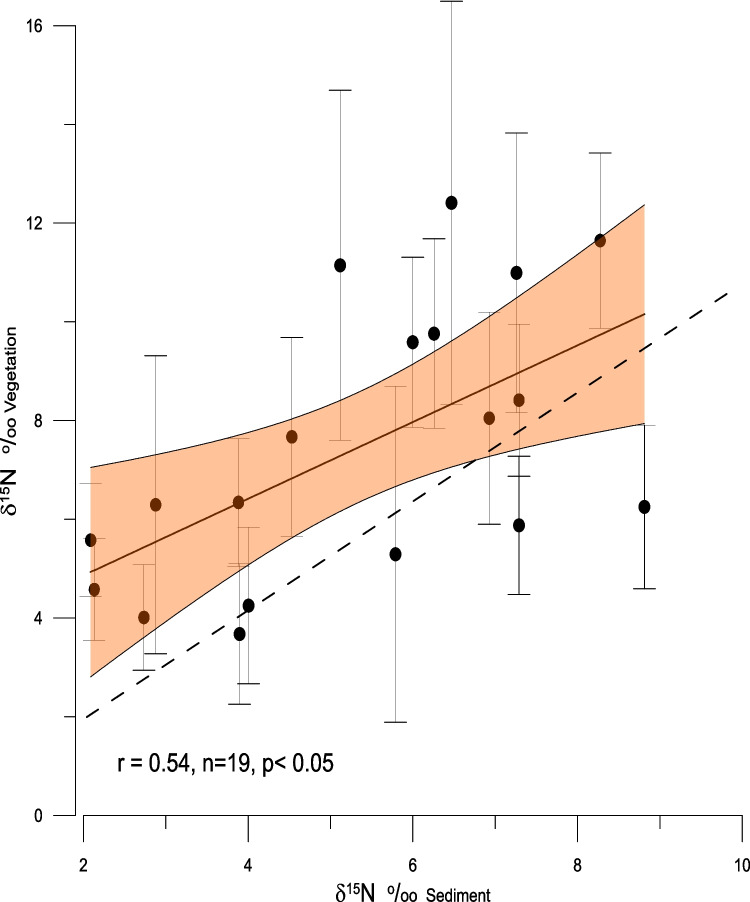
The relationship between the δ^15^N_SED_ values and the mean δ^15^N_VEG_ values for the 19 sites in which the δ^15^N value of the sediment was measured. The dashed line represents a 1:1 relationship between the δ^15^N_SED_ and δ.^15^N_VEG_. The spearman rank correlation coefficient is 0.565 (*p* < 0.05). The best fit line shows that the vegetation is approximately 3‰ more positive than the sediments; See Fig. 4

The average δ^15^N value of the sedimentary organic material (δ^15^N_SOM_) is + 4.96 (± 1.76) and showed a positive correlation with the δ^15^N_veg_ values during all sampling months (r = 0.61, *n* = 21 *p* < 0.05). Values for all sites are given in Table 3 and in Figs. 4 and 5. In most instances the δ^15^N_SOM_ values were lower than the δ^15^N_veg_ values from the same site, with an average difference of 3.2‰ (Fig. 5). The spatial pattern of δ^15^N_SOM_ values are shown in supplemental material Figure S4-15.

**Table 3:** Mean δ^15^N values and standard deviations of organic material in sediments

	δ^15^N	sd	n
	‰	‰	
AC1	1.97	1.51	2
AC2			0
BCE	9.34		1
BCW	2.98		2
BB	4.53	1.54	2
EGP	5.44	1.70	4
JMP	6.34	0.21	4
KP	2.88		1
KBBC	4.00		1
KBYC	2.09		1
LR1	6.26		1
LR2	3.94	2.59	5
LRPH	5.56	0.20	2
LRPP	6.60	0.58	3
MD	2.13		1
MH	4.67	3.32	4
MCD	5.75	0.22	3
MMS	2.73		1
MS	7.14	0.49	3
RSMAES	6.16	3.57	3
SAL	3.88		1
SSBR	4.65	3.89	3

### Dissolved inorganic nitrogen

**Fig. 6:**
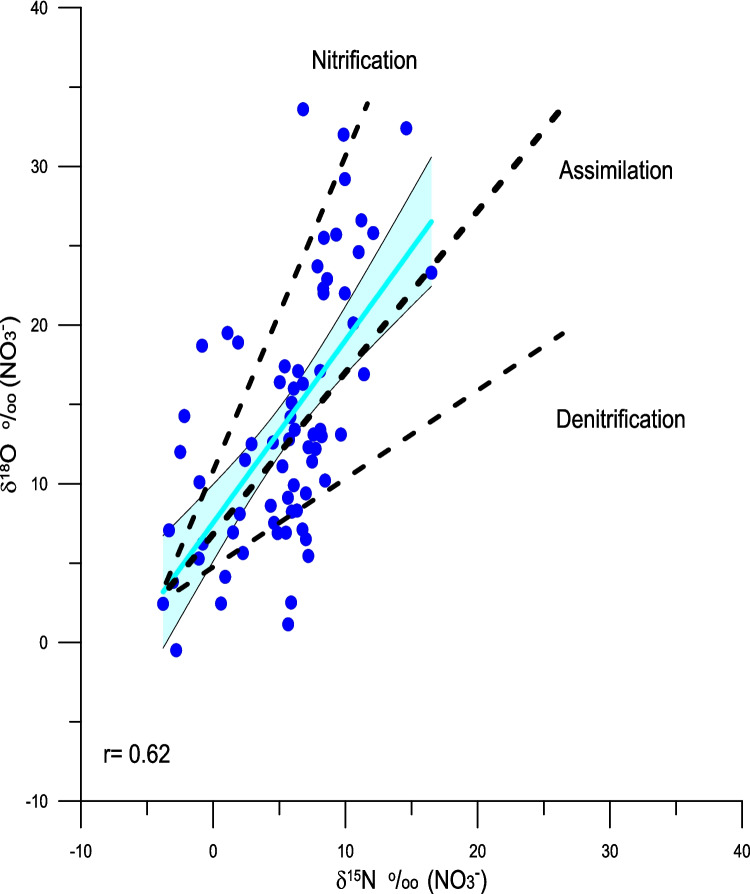
Scatter plot between the δ^15^N_NO3_ and δ^18^O_NO3_ values (r = 0.62, *n* = 70, *p* < 0.01). The shaded area represents the 95% confidence limits of the fit of the regression line. Also indicted are the expected relationships between δ^15^N_NO3_ and δ^18^O_NO3_ values for the process of nitrification (2:1), assimilation (1:1), and denitrification (1:2)

**Fig. 7:**
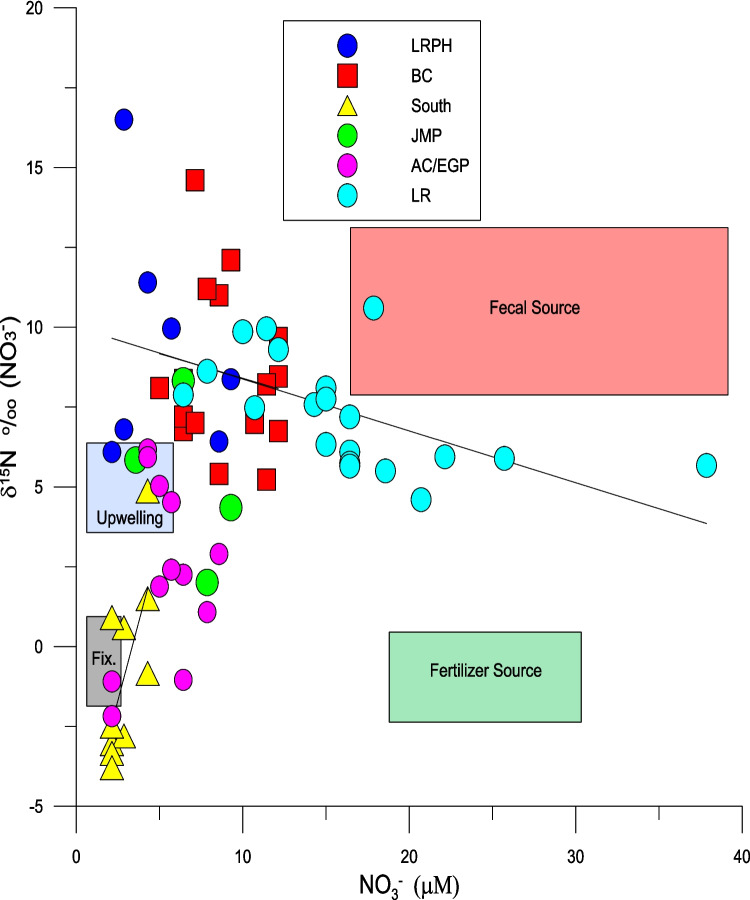
Scatter plot of δ^15^N_NO3_ values and NO_3_^−^ concentrations differentiated on the basis of localities (r = 0.3, *n* = 70, *p* < 0.05) While the relationship is statistically significant, it only explains 10% of the variance. This low variance is a result of different trends at other sites. For example, sites from the Little River (LR) consistently show an inverse correlation between δ^15^N_NO3_ values and NO_3_^−^concentration (r = −0.75, *n* = 27, *p* < 0.05), interpreted as reflecting fractionation during assimilation. Other sites exhibit a positive correlation, probably resulting from mixing between different sources of nitrogen. Theoretical range of sources of the δ.^15^N_NO3_ values have been taken from Kendall et al. (2008)

While the low concentration of nitrate (< 1.0 μM) at the majority of the marine sites generally precluded the measurement of δ^15^N_NO3_ and δ^18^O_NO3_ values, concentrations were sufficient at Little River, Biscayne Canal, Arch Creek, East Greynolds Park, and Jose Marti Park, all canal sites draining into North Biscayne Bay. On a few occasions (6 out of 70 samples), sites in the southern portion of the study area had sufficient NO_3_^−^ to measure the δ^18^O and δ^15^N values (Table 1). These sites had much lower δ^15^N_NO3_ and δ^18^O_NO3_ values compared to those in the north and showed no statistically significant correlation between the δ^15^N_NO3_ and δ^18^O_NO3_ values. The δ^15^N_NO3_ and δ^18^O_NO3_^–^ values of all the samples analyzed are shown in Fig. 6 and Table 4 and the δ^15^N_NO3_ values relative to the concentration of NO_3_^−^ in Fig. 7. The δ^15^N_NO3_ values range between −3.79 and + 16.5‰ with a mean of + 5.54‰ and the δ^18^O values range between −0.49‰ and + 33.6‰ with a mean of + 13.91‰. The δ^15^N_NO3_ and δ^18^O_NO3_ values are positively correlated (r = 0.62, *n* = 70, *p* < 0.05) (Fig. 6). Considering the dataset in its entirety, changes in the concentration of NO_3_^−^ are weakly correlated with δ^15^N_NO3_ (r = 0.3, *n* = 70, *p* < 0.05) values (Fig. 7) and not statistically significantly correlated with δ^18^O_NO3_ (r = 0.173, *n* = 70, *p* > 0.05).

**Table 4:** The δ^18^O and δ^15^N values and concentration of NO_3_^−^ of water samples analyzed in this study

Date	ID	δ^18^O	δ^15^N	NO_3_^−^	Date	ID	δ^18^O	δ^15^N	µM NO_3_^−^
		‰	‰	µM			‰	‰	µM
6/15/2022	AC 1	19.50	1.08	7.86	11/3/2021	LR1	25.70	9.30	12.14
6/15/2022	AC 2	5.63	2.25	6.43	12/8/2021	LR1	20.10	10.60	17.86
7/9/2021	AC1	18.90	1.88	5.00	6/6/2022	LR1	5.45	7.19	16.43
6/6/2022	AC1	12.60	4.52	5.71	6/10/2022	LR1	9.12	5.65	16.43
7/9/2021	AC2	10.10	−1.04	6.43	6/15/2022	LR1	7.53	4.60	20.71
10/4/2021	AC2	5.28	−1.09	2.14	7/9/2021	LR2	9.91	6.09	16.43
11/3/2021	AC2	14.27	−2.18	2.14	10/6/2021	LR2	12.80	5.74	16.43
6/6/2022	AC2	12.50	2.90	8.57	11/3/2021	LR2	32.00	9.86	10.00
6/13/2022	BB	6.23	−0.79	2.14	12/8/2021	LR2	22.00	9.95	11.43
7/9/2021	BCE	16.30	6.78	6.43	6/6/2022	LR2	2.52	5.89	25.71
10/4/2021	BCE	10.20	8.46	12.14	6/10/2022	LR2	8.24	5.94	22.14
11/3/2021	BCE	25.80	12.10	9.29	6/15/2022	LR2	1.14	5.67	37.86
6/6/2022	BCE	12.30	7.20	6.43	7/9/2021	LRPH	16.00	6.09	2.14
6/15/2022	BCE	6.51	7.01	10.71	10/6/2021	LRPH	17.10	6.42	8.57
7/9/2021	BCW	22.00	8.34	6.43	11/3/2021	LRPH	29.20	9.96	5.71
10/6/2021	BCW	13.10	9.66	12.14	12/8/2021	LRPH	25.50	8.37	9.29
11/3/2021	BCW	26.60	11.20	7.86	6/6/2022	LRPH	33.60	6.80	2.86
12/8/2021	BCW	32.40	14.60	7.14	6/10/2022	LRPH	23.30	16.50	2.86
6/6/2022	BCW	7.12	6.75	12.14	6/15/2022	LRPH	16.90	11.40	4.29
6/15/2022	BCW	9.39	7.01	7.14	7/28/2021	LRPP	13.40	8.10	15.00
7/9/2021	EGP	13.40	6.17	4.29	10/6/2021	LRPP	11.40	7.48	10.71
10/4/2021	EGP	11.50	2.41	5.71	11/3/2021	LRPP	23.70	7.88	6.43
6/6/2022	EGP	15.10	5.93	4.29	12/8/2021	LRPP	22.90	8.62	7.86
6/15/2022	EGP	16.40	5.03	5.00	6/6/2022	LRPP	8.31	6.33	15.00
11/1/2021	JMP	22.30	8.32	6.43	6/15/2022	LRPP	6.92	5.50	18.57
12/6/2021	JMP	14.21	5.84	3.57	7/28/2021	MCDS	17.40	5.41	8.57
6/13/2022	JMP	8.62	4.35	9.29	10/4/2021	MCDS	13.00	8.21	11.43
7/7/2021	JMP	8.10	2.01	7.86	11/3/2021	MCDS	24.60	11.00	8.57
6/13/2022	KBBC	7.07	−3.34	2.14	6/6/2022	MCDS	17.10	8.09	5.00
6/13/2022	KBYC	6.89	4.87	4.29	6/15/2022	MCDS	11.10	5.23	11.43
7/7/2021	KP	12.00	−2.49	2.14	7/7/2021	MD	18.70	−0.84	4.29
6/13/2022	KP	2.43	−3.79	2.14	6/8/2022	MD	2.45	0.59	2.86
7/9/2021	LR1	13.10	7.58	14.29	6/13/2022	MMS	3.82	−3.04	2.14
10/6/2021	LR1	12.20	7.75	15.00	6/13/2022	RSMAES	−0.49	−2.80	2.86

### Correlation between δ^15^N_NO3_ and δ^15^Nveg values

**Fig. 8:**
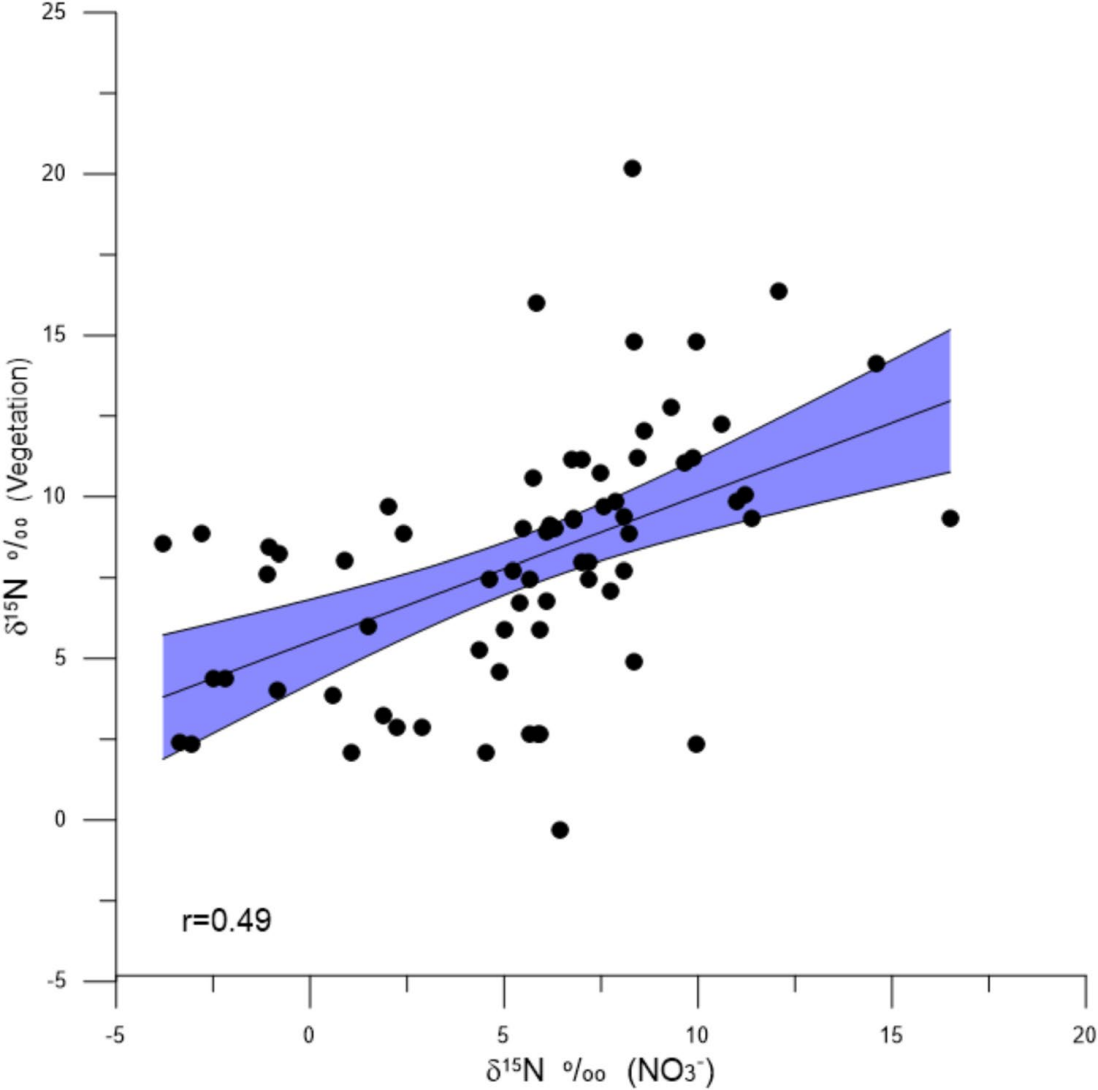
Scatter plot of δ^15^N_NO3_ and δ.^15^N_veg_. Overall regression coefficient is + 0.49 (*p* <  < 0.01, *n* = 70)

The δ^15^N_NO3_ and δ^15^N_veg_ values show a positive correlation of + 0.50, (*p* < 0.05) (Fig. 8), with a slope of 0.45, so that at low δ^15^N_NO3_ values, the δ^15^N_veg_ values are higher than those of the NO_3_^−^ while at higher δ^15^N_NO3_ values, the δ^15^N_veg_ values are lower.

### Correlation with other environmental parameters

Correlation coefficients were calculated between the δ^15^N_VEG_ and the other chemical and biological parameters measured during this study on a site-by-site basis, over annual and monthly timescales. The regression coefficients between all parameters for the annual samples are shown in Table 5.

**Table 5:** Correlation between annual mean values of water quality parameters measured at all 22 sites relative to the N isotopic composition of vegetation and organic material in sediments; DO = dissolved oxygen, S = salinity, T = temperature, CH = chlorophyll, MPN = enterococci, TN = total nitrogen, TP = total phosphorus, N&N = Total NOx, NO_3_ = nitrate, NH_4_ = ammonia, SRP = soluble reactive phosphorus, DOC = dissolved organic carbon, δ^15^N = δ^15^N value of vegetation, δ^15^N s = δ^15^N value of sediment, H = Human HF 183 genetic marker, D1 = the “DogBact” Taqman assay for dog host specific fecal *Bacteroides* bacteria, D2 = the “DG3” Taqman assay for dog host specific fecal *Bacteroides* bacteria. Highlighted values are statistically significant at the 95% confidence limits. Similar analyses for individual months and stations are presented in the supplemental material. As a result of the relatively small number of water samples analyzed for their δ^15^N and δ^18^O values, no correlation coefficients are shown for these parameters

	DO	S	T	CH	MPN	TN	TP	N&N	NO3	NH4	SRP	DOC	δ15N	δ15N s	H	D1	D2
Temp (℃)	−0.41	−0.66	−0.15	0.13	0.68	0.63	0.42	0.57	0.56	0.43	0.40	0.63	0.33	0.31	−0.08	0.02	0.20
DO (mg/L)		0.46	0.14	−0.27	−0.67	−0.66	−0.49	−0.67	−0.67	−0.58	−0.53	−0.37	−0.10	−0.10	−0.11	−0.34	−0.27
Salinity (ppt)			0.24	−0.48	−0.61	−0.81	−0.47	−0.80	−0.79	−0.50	−0.35	−0.94	−0.57	−0.21	0.17	−0.03	−0.06
Turbidity (NTU)				0.18	−0.24	−0.07	0.26	−0.25	−0.25	−0.04	0.04	−0.31	−0.47	−0.15	−0.04	−0.21	−0.17
Chlorophyll (RFU)					0.35	0.46	0.47	0.15	0.13	0.45	0.29	0.29	−0.12	0.00	−0.11	−0.12	−0.19
MPN (/100 ml)						0.82	0.68	0.60	0.59	0.80	0.74	0.49	0.12	0.09	−0.23	0.09	0.01
TN (µM)							0.84	0.69	0.67	0.84	0.76	0.70	0.22	−0.01	−0.09	0.00	−0.06
TP (µM)								0.28	0.26	0.93	0.93	0.26	−0.23	−0.19	−0.05	−0.06	−0.15
N&N (µM)									1.00	0.33	0.26	0.78	0.64	0.30	−0.17	0.10	0.13
NO3 (µM)										0.31	0.23	0.78	0.65	0.31	−0.17	0.10	0.14
NH4 (µM)											0.96	0.26	−0.16	−0.18	−0.10	−0.02	−0.11
SRP (µM)												0.14	−0.20	−0.18	−0.06	−0.02	−0.11
DOC (µM)													0.70	0.26	−0.10	0.06	0.09
δ^15^N veg														0.58	−0.05	0.19	0.31
δ^15^N sed															−0.11	0.33	0.55
H																−0.10	−0.08
D1																	0.90

#### Annual

On an annual basis most of the measured parameters (T, DO, S, Chl., Enterococci, TN, NO_3_^−^, NH_4_^+^, SRP, and DOC) did not show any statistically significant correlations with δ^15^N_VEG_ or δ^15^N_SED__,_ (Table 5). However, five of the variables showed statistically significant relationships with δ^15^N_VEG_ values. One variable, turbidity, showed statistically significant relationships with the CN ratio.

The δ^15^N_VEG_ values were inversely correlated with salinity and turbidity (< 0.05, *n* = 22) and there was a positive correlation between δ^15^N_VEG_ values and dissolved organic carbon (*p* < 0.05). The δ^15^N_VEG_ values were positively correlated with the concentration of NO_3_^−^ and NO_3_^−^ + NO_2_^−^ (NO_x_).

#### Monthly

Examination of the same patterns within individual months showed rather poor relationships and did not replicate the trends evident in the annual data.

#### Septic systems

The number of septic tanks were calculated within 5, 4, 3, 2, and 1 km radius of each site (Silverstein et al. 2024) and the correlation between the number of tanks and the δ^15^N_VEG_ values determined. While none of the correlation coefficients was statistically significant there was always a weak positive correlation between the number of septic systems and the annual average δ^15^N_VEG_, with the highest correlation coefficient (0.43) occurring when the number of septic systems within a 3 km radius was considered. Using this radius, the correlations were investigated on a monthly basis with statistically significant correlations (< 0.05) only present in January 2022 and May 2022.

#### Population density

Population density was positively correlated with annual δ^15^N_VEG_ values at intervals of 5, 4, 3, and 2 km radius (r > 0.6 to 0.7, *p* < 0.05). Similar patterns were observed for every month of the study.

#### Rainfall and flow through canals

The amount of precipitation and water flow through the canals was calculated and correlated to the δ^15^N_VEG_ values. Both these variables showed an inverse correlation with δ^15^N_VEG_ values. For water flow r = −0.55 (*p* < 0.05) over periods of between 8 and 12 months and rainfall r = −0.57 (*p* < 0.05) for periods of between 5–6 months.

## Discussion

### Nitrogen isotopes as an indicator of sewage

This study has shown substantially elevated δ^15^N values of plant material and NO_3_^−^ sampled from canals draining into northern Biscayne Bay. Such high δ^15^N values have been traditionally interpreted as reflecting sewage input (Aravena et al. 1993; Costanzo et al. 2001; Heaton 1986; Sammarco and Strychar 2013) and the evidence from this study certainly shows that the region in this study is contaminated from this source. The areas with the highest δ^15^N values (Biscayne Canal, Little River, and Miami River), are known to be neighborhoods with abundant septic tanks and having the highest instances of septic tank failures (Miami Dade Water and Sewar Department 2020). In contrast, the δ^15^N values from the marine environments (Key Biscayne, Coconut Grove, and Matheson Hammock) generally show lower values + 2 to + 5‰), similar to those measured in *Sargassum natans*, a known pelagic species believed to originate in the Atlantic and/or Caribbean. The benthic variety of sargassum, *Sargassum pteropleuron*, found in Biscayne Bay close to the coastline, has a higher δ^15^N values (+ 6 to + 8‰) than the pelagic variety. While the mean δ^15^N values in the marine environments are lower than those measured in the canals, there are instances in which quite positive δ^15^N values were measured within some of the green (*Halimeda* sp.) and brown algae (*Padina* sp*., Dictyota* sp.*,* and *Sargassum pteropleuron*) particularly at the Matheson Hammock and Coconut Grove sites indicating some influence from water elevated in ^15^N. The δ^15^N_NO3_ values are positively correlated with the δ^15^N_veg_ values (+ 0.50, *n* = 70, *p* < 0.05) (Fig. 8). The absence of a stronger correlation between the δ^15^N_veg_ and δ^15^N_NO3_ values reflects the fact that the δ^15^N_veg_ values integrates the δ^15^N_NO3_ values over a growth period of several weeks, while the δ^15^N_NO3_ value represents the value at the time of collection. The δ^15^N_NO3_ values probably vary substantially over the growth period of the vegetation as a result of rainfall and tidal processes.

### Interpretation and modeling of trends in the δ^15^N values

**Fig. 9:**
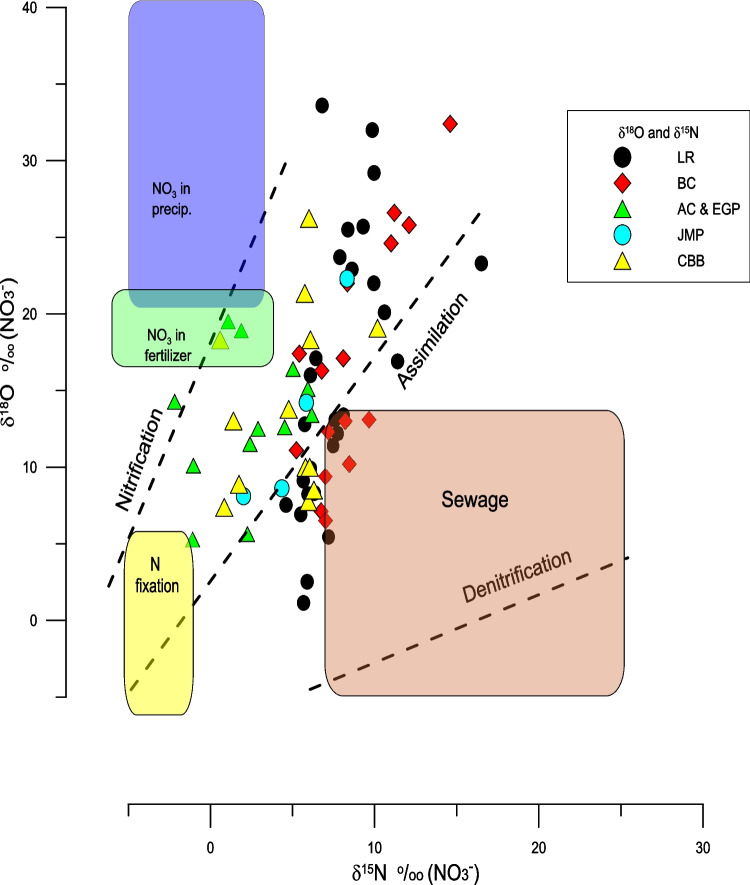
The δ^15^N_NO3_ and δ^18^O_NO3_ values reflect processes of assimilation and nitrification as well as and mixing with NO_3_^−^ derived from fertilizer and precipitation. The δ^15^N_NO3_ and δ^18^O_NO3_ values for Central Biscayne Bay (CBB) have been taken from Swart et al. (2013). Ranges of the δ^15^N_NO3_ and δ.^18^O_NO3_ values have been taken from Kendall et al. (2008)

**Fig. 10:**
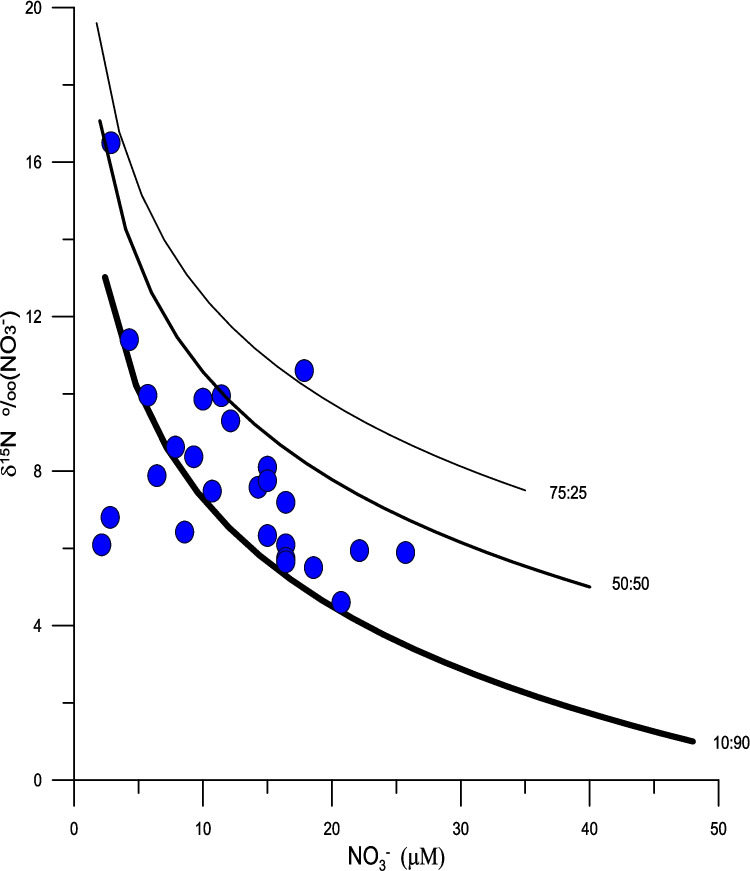
Scatter plot of δ^15^N_NO3_ values and NO_3_^−^ concentrations from Little River plotted with the output from a combined mixing between sewage and inorganic fertilizers in ratios of 10:90, 50:50, and 75:25 followed by Rayleigh distillation that predicts the evolution of the δ^15^N_NO3_ values as function of fractionation during assimilation of NO_3_^−^ by plants using an α value of 1.004 (Swart et al. 2014) as the concentration of NO_3_^−^ decreases. While the best fit to the data appears to be with an initial mixture of approximately 50:50, the systems are in reality extremely complex with many inputs to such a model unknown

While the spatial patterns of the δ^15^N_veg_ values are clearly influenced by anthropogenic sources, the δ^15^N_veg_ and δ^15^N_NO3_ values are in some instances higher (Fig. 6, 7, and 10) than would be expected from a human source of between + 8 and + 10‰ (Bowen et al. 2009). Such elevated values are probably a result of fractionation during assimilation or during partial nitrification and volatilization of NH_4_^+^ that result in elevated δ^15^N values of NO_3_^−^ and NH_4_^+^. Fractionation during these processes can be tracked both by the nature of correlation between the concentration of NO_3_^−^ and δ^15^N_NO3_ values (Fig. 7) and δ^15^N_NO3_ and δ^18^O_NO3_ values (Fig. 9) (Aravena and Robertson 1998). Considering all sites together there is a weak positive relationship between the concentrations of NO_3_^−^ and their δ^15^N_NO3_ values (r = 0.3, n = 70, *p* < 0.05). A positive correlation might be expected as a result of simple mixing between natural sources of nitrate with a δ^15^N value close to zero and a sewage source with an elevated δ^15^N value. However, based on visual inspection it is evident that there is a wide range of δ^15^N_NO3_ values at low concentrations of NO_3_^−^, suggesting that there are other processes at play. If the Little River sites are considered separately, there is a statistically significant inverse correlation between the concentration of NO_3_^−^ and δ^15^N_NO3_ values (r = −0.51, n = 27, *p* < 0.01), while all of the other sites show a positive correlation between NO_3_^−^ and δ^15^N_NO3_ values (r =  + 0.64, n = 43, *p* < 0.01) (Fig. 9). The explanation for the difference between these two groups is that fractionation during assimilation of NO_3_^−^ is greatest in areas, such as Little River, where the concentrations of NO_3_^−^ are highest. Here the canal drains through a disused golf course on which inorganic fertilizers had been previously applied. Therefore, the water in this canal is a mixture between nitrate derived from artificial fertilizer and sewage. Here, a two-step model can be applied. First, the initial starting concentration of NO_3_^−^ and δ^15^N values of + 6 ‰ can be estimated by mixing fertilizer and sewage derived nitrogen. In the example shown in Fig. 10 percentages of between 75 and 10% sewage derived N with a δ^15^N values of + 10 ‰ has been mixed with fertilizer nitrogen with a δ^15^N values of 0‰. Using these starting compositions, Rayleigh distillation models, using an α value of 1.004 (as reported by Swart et al. 2014), predict the evolution of the values as shown in Fig. 10. Considering the numerous uncertainties in this model, the ranges measured agree reasonably well using a mixture of 60% fertilizer and 40% sewage nitrogen. In contrast, the trends present at the other sites might be better explained by mixing between sewage derived NH_4_^+^ and NO_3_^−^, and N originating either from artificial fertilizers and/or nitrogen fixation.

### Interpretation of the range of δ^18^O values

The slope of the relationship between the δ^15^N_NO3_ and δ^18^O_NO3_ values also provides evidence that processes such as assimilation and nitrification are important in explaining the stable N and O isotope patterns (Fig. 9). Assimilation generally produces a 1:1 slope between the δ^15^N_NO3_ and δ^18^O_NO3_ values, while nitrification results in a slope closer to 2:1. As the actual magnitude of the slope measured in this study is 1.14, it is possible that both processes may be influential. As seen in Fig. 9, the δ^15^N_NO3_ and δ^18^O_NO3_ values of the samples analyzed in this study do not fall within the sewage domain as postulated by Kendall et al. (2008), but rather suggest the importance of atmospheric NO_3_^−^ delivered by rainfall and artificial fertilizers (Elliott et al. 2007). Similar conclusions have been derived from the study of other urban watersheds (Yang and Toor 2016). In that study, based on measurement of δ^15^N_NO3_ values in the canals and rainfall, it was suggested that as much as 70% of the NO_x_ load could be derived from atmospheric sources. Considering that the area of Miami investigated in this study is heavily urbanized and subject to a significant amount of vehicular traffic, a greater contribution from this source is possible. While the concentrations of atmospheric NO_x_ measured in Miami (20–40 ppb) by the Department of Environmental Protection, are well below the EPA limit of an average of 100 ppb over a period of an hour, it may be that wet deposition of NOx derived from combustion engines is a significant source and this should be confirmed by flux of N in precipitation and its δ^15^N_NO3_ value.

### Temporal variation of δ^15^N values

There have been several studies that have proposed that the δ^15^N values of vegetative components reach a maximum value during the wet season (Lapointe et al. 2004, 2019) as it has been argued that this is the time when most anthropogenic derived materials are washed into the canals and subsequently into the adjacent marine environments. However, critical examination of such studies reveal that the majority were not conducted at a sufficiently high enough temporal resolution to make this case. In this study, where there is clear evidence that there is contamination from sewage derived components, the highest δ^15^N values are present in vegetation collected at the end of the wet season in Biscayne Canal and Jose Marti Park and in the dry season in Little River (Fig. 4). This is shown by the inverse correlations between rainfall/water flow and the δ^15^N_VEG_ values. At the marine sites, the highest values are found in the dry season. The rationale for this is twofold. First, the input of sewage derived nutrients continuously, regardless of the amount of rainfall. While increased rainfall may bring more sewage derived nutrients into the canals, it also dilutes the water and moves it into Biscayne Bay. While such reasoning would predict higher δ^15^N values in the marine environments during the wet season, which differences are not evident in this study (Fig. 4).

### Sedimentary δ^15^N values

At the start of this study, it was believed that the sedimentary organic material might be a good archive of the mean δ^15^N values of the biota found at the various sites. However, while there is a statistically significant correlation between the δ^15^N_SED_ and the δ^15^N_VEG_ values (*p* < 0.05), the correlation is perhaps not as robust as one might expect and the sedimentary δ^15^N values are about 3‰ lower than those of the live vegetation collected at each site (Figs. 4 and 5). This mismatch is probably a result of at least two factors. First, the organic material found in the sediment at any canal site is not only a reflection of material derived from the immediate environment, but from a wide range of localities, not only upstream and downstream, but also from the adjacent land mass. The marine sites may also suffer from input of material that is not local, but probably to a lesser extent than the canals. A second factor contributing to the more negative values of the δ^15^N_SED_, is preferential degradation of organic compounds, such as amino acids, known to have elevated δ^15^N values when compared to the bulk values (Hassan et al. 2025; McClelland and Montoya 2002). Typically those amino acids with the more positive δ^15^N values, relative to the bulk composition, degrade easily during early oxidation of the organic material (Lehmann et al. 2020), leaving the δ^15^N_SED_ more negative than the original organic material. The extent of this effect is obviously variable depending upon the nature of the organic material that originally contributed to the sediment.

### Correlation with other water quality parameters

The correlations between δ^15^N_VEG_ values and other water quality parameters measured on water samples collected at the same time are shown in Table 5. While there are statistically significant correlations between δ^15^N_VEG_ values and concentrations of NO_3_^−^ and NH_4_^+^, there are no statistically significant correlations with fecal indicators such as the concentration of Enterococci and the human HF 183 fecal Bacteroides MST marker. The absence of correlation with human fecal markers is perhaps not surprising considering that the vegetation samples represent material produced over periods from days to weeks, whereas the nutrients and bacteria concentrations represent instantaneous measurements. This difference is exemplified by differences in the response of the δ^15^N_VEG_ values and other indices to changes in precipitation. The δ^15^N_VEG_ values reached a peak in the dry season, while the concentration of nutrients and fecal indicators are higher in the wet season. The rainfall events clearly flush the nutrients and fecal indictors rapidly into the canals and the marine waters, while in the dry season the nutrients linger longer in the canals. This not only allows more time for assimilation of the nutrients, but also for processes such as assimilation and nitrification to fractionate the isotopic composition. Interestingly there is a positive correlation between the δ^15^N_SED_ values and one of the genetic markers for canine waste. Presumably the sediments integrate both the canine biomarkers and the δ^15^N values over longer time periods, thus producing the observed correlation. These differences are also seen in sites such as Arch Creek which consistently had the highest concentrations of nutrients, levels of Enterococci and genetic fecal markers. In contrast the δ^15^N_VEG_ values at this site, while high (~ + 6‰), were much lower than found at other sites such as Little River, Biscayne Canal, and Miami River (Fig. 7 and 9) that had lower concentrations of Enterococci and genetic fecal markers. The variables that were correlated with δ^15^N_VEG_ values, turbidity, salinity, DIN, NO_3_^−^, and TOC are more or less permanent features of the particular environment and are present throughout the year, so broad correlation between these variables and the δ^15^N_VEG_ values is expected.

### Septic tank density and human population

While there is a statistically significant positive correlation between δ^15^N_VEG_ values and population density, there was no correlation between the number of septic systems and population density, and therefore no correlation between septic tank density and δ^15^N_VEG_ values. This finding probably speaks to the complicated relationship between the δ^15^N value of the effluent from septic system and the condition of the septic systems, several aspects of which are still not fully resolved.

In a well aerated and maintained septic system, organic material is decomposed and NH_4_^+^ released into the water in the septic tank. Here some oxidation of the NH_4_^+^ to NO_3_^−^ occurs, causing enrichment of ^15^N in the NH_4_^+^ and depletion of ^15^N in the NO_3_^−^. Eventually these fluids pass into the adjacent drain field where the NH_4_^+^ is oxidized to NO_3_^−^ (Wilhelm et al. 1994a). Water from the drain field leaches into adjacent areas where it can be partially utilized by plants, with excess NO_3_^−^- rich water eventually draining into waterways and ultimately Biscayne Bay. While under such conditions, the δ^15^N values of the NO_3_^−^ should be a composite of the δ^15^N values of the solid material and urea that entered into the septic tanks., even in well maintained systems there may be a relatively poor supply of oxygen leading to limited anaerobic conditions and hence denitrification. In this process NO_3_^−^ is used by microbes as a terminal electron acceptor to oxidize organic material, leading to further enrichment of the δ^15^N values above the original values of the sewage an thus reducing the concentration of NO_3_^−^. While there have not been extensive studies of the δ^15^N values of the various nitrogen species produced in septic systems in different states of repair, one might expect a fully functioning system to produce NO_3_^−^ with δ^15^N values similar to the values of the input. As the areas around the drain field would not be subject to clogging and anoxia, such areas might be expected to support a healthy plant life that would in turn utilize the high δ^15^N values in the soil and lead to the eventual consumption of the majority of the available nitrate arising from the system. Under conditions of oxygen starvation, caused by a poorly aerated drain field, NH_4_^+^ is not converted to NO_3_^−^ and concentrations of dissolved organic carbon and NH_4_^+^ form in the groundwater causing clogging and limiting the effluent drainage (Wilhelm et al. 1994a, 1994b). Such flow could be further impeded by a high-water table combined with saltwater intrusion Miami Dade Water and Sewar Department (2020). Under such condition anaerobic oxidation of NH_4_^+^ (Anammox) would lead to elevation of the ^15^N of the NH_4_^+^ (Brunner et al. 2013). Water containing nitrogen species would leak into canals where the residual ^15^N-rich NH_4_^+^ would be oxidized to NO_3_^−^ and both species assimilated by plants thus leading to δ^15^N values in excess of those originally in the sewage. Leakage from septic tanks might be enhanced during low tide or high rainfall events. In fact, isotopic fractionation caused by nitrification and assimilation may be a major process as indicated by the slope between the δ^15^N_NO3_ and δ^18^O_NO3_ values that lies between the theoretical slope of 2:1 for nitrification and 1:1 for assimilation (Figs. 6 and 9). Such results have been observed in storm-water biofiltration studies where the biofilter was a net producer of nitrate via nitrification. Feraud et al. (2023) showed biofilter soil core eluates that were found to exhibit a δ^15^N_NO3_ vs δ^18^O_NO3_ slope of ~ 1.8, consistent with multiple fractionation pathways. That study also found that NH_4_^+^ absorbs and nitrifies during biofilter dry-down and then is flushed out of the system during storm events as nitrate. The absence of such correlation may be because a significant amount of the increase in the δ^15^N values takes place once the NO_3_^−^ is in the canals during the processes of nitrification and assimilation. As the water in the canal then rapidly moves into the coastal bays according to the water flow, precipitation, and tidal state, concentrations of nitrate and its δ^15^N value may be unrelated to the septic tank density.

## Conclusions

- This study represents one of the more comprehensive investigations into the use of δ^15^N values in both vegetation and nitrate to understand both the sources and processes affecting anthropogenic derived nutrients in a heavily urbanized coastal environment. In this study, canals and waterways passing through urban areas in northern Miami-Dade County, where there are abundant septic systems and a large proportion are in disrepair, exhibit consistently very positive δ^15^N values (+ 8 to + 20‰) in the vegetation and NO_3_^−^ values. These values do not reach a maximum in the wet season, but rather at the end of the wet season and well into the dry season. While the δ^15^N values were much lower in the marine environments, there were some instances of values higher than normal values at these locations too. However, since the actual extent of enrichment in ^15^N in DIN is influenced by fractionation during assimilation of DIN by micro and macro organisms as well as during nitrification (Casciotti et al. 2003; Delwiche and Steyn 1970), and denitrification (Barford et al. 1999; Granger et al. 2006; Miyake and Wada 1971), it is not practical to assign a precise value for δ^15^N values which would unequivocally indicate the presence of sewage. At low concentrations of nitrate, typical of open marine environments, all the available nitrate is rapidly consumed and hence there is no fractionation associated with assimilation. In the Atlantic Ocean the DIN supplied by upwelling has a δ^15^N value ~  + 5‰ and because concentrations are typically low, the plankton assimilating this DIN will have approximately the same δ^15^N value. However, if there are high concentrations of NO_3_^−^, typical of the canals feeding northern Biscayne Bay, fractionation takes place and the δ^15^N values of the algae or plant material are slightly less than that in the environment. As the parcel of water moves away from the source, the concentration of NO_3_^−^ decreases and the δ^15^N value increases. The elevation of the δ^15^N values greatly in excess of those typical of humans, speaks to this process. Therefore, while high δ^15^N values (> + 10‰) generally suggest the presence of nitrogen derived from the activities of higher trophic organisms (Heaton 1986), high values can be produced by assimilation, even if the initial δ^15^N value is low. Therefore, the contention that values between + 3‰ and + 5‰ reflect the influence of anthropogenic sewage is not in any way realistic. Not only are such values typical of the normal marine environment, but they can be easily produced as a result of Rayleigh fractionation during the assimilation even if the original δ^15^N value is low. One example of such an environment might be an area in which artificial fertilizers are liberally used, such as on golf courses and in areas of intensive agriculture.
- Additional contributions to the NO_3_^−^ budget might include atmospheric contributions from internal combustion engines and Saharan dust, although such contributions need to be verified by a combination of the measurements of concentrations and isotopic studies of local precipitation.

## Supplementary Information

Below is the link to the electronic supplementary material.

Supplementary file1 (RAR 10559 KB)

## Data Availability

All data contained in this paper are included in the supplemental material and archived in the Earthchem database (10.60520/IEDA/113788).
